# The multifaceted role of Fragile X-Related Protein 1 (FXR1) in cellular processes: an updated review on cancer and clinical applications

**DOI:** 10.1038/s41419-023-06413-8

**Published:** 2024-01-18

**Authors:** Faiz Ali Khan, Na Fang, Weijuan Zhang, Shaoping Ji

**Affiliations:** 1 Huaihe Hospital，Medical School, Henan University, Kaifeng, China; 2 Laboratory of Cell Signal Transduction, Department of Biochemistry and Molecular Biology, School of Basic Medical Sciences, Henan University, Kaifeng, China; 3 Department of Basic Sciences Research, Shaukat Khanum Memorial Cancer Hospital and Research Centre (SKMCH&RC), Lahore, Pakistan; 4 Zhengzhou Shuqing Medical College, Zhengzhou, China

**Keywords:** Gene silencing, Ubiquitylation, Diagnostic markers, Ubiquitylation

## Abstract

RNA-binding proteins (RBPs) modulate the expression level of several target RNAs (such as mRNAs) post-transcriptionally through interactions with unique binding sites in the 3′-untranslated region. There is mounting information that suggests RBP dysregulation plays a significant role in carcinogenesis. However, the function of FMR1 autosomal homolog 1(FXR1) in malignancies is just beginning to be unveiled. Due to the diversity of their RNA-binding domains and functional adaptability, FXR1 can regulate diverse transcript processing. Changes in FXR1 interaction with RNA networks have been linked to the emergence of cancer, although the theoretical framework defining these alterations in interaction is insufficient. Alteration in FXR1 expression or localization has been linked to the mRNAs of cancer suppressor genes, cancer-causing genes, and genes involved in genomic expression stability. In particular, FXR1-mediated gene regulation involves in several cellular phenomena related to cancer growth, metastasis, epithelial-mesenchymal transition, senescence, apoptosis, and angiogenesis. FXR1 dysregulation has been implicated in diverse cancer types, suggesting its diagnostic and therapeutic potential. However, the molecular mechanisms and biological effects of FXR1 regulation in cancer have yet to be understood. This review highlights the current knowledge of FXR1 expression and function in various cancer situations, emphasizing its functional variety and complexity. We further address the challenges and opportunities of targeting FXR1 for cancer diagnosis and treatment and propose future directions for FXR1 research in oncology. This work intends to provide an in-depth review of FXR1 as an emerging oncotarget with multiple roles and implications in cancer biology and therapy.

## Facts

- FXR1s modulate RNA metabolism and are correlated with the development of malignancy.
- Alteration in FXR1 expression and function enhance the activities of cancer driver genes, promote tumor development, and trigger malignant behavior.
- New technologies, in conjunction with genetically engineered animal models, are assisting in the discovery of FXR1 molecular pathways in cancer.
- Understanding the FXR1 functions in cancer cells will help develop biomarkers for prognosis, as well as possibly reveal new targets for the design of therapies.

## Open questions

- What role does FXR1 play in cancer progression through the regulation of RNA stability?

- How to decipher the network of intricate connections between FXR1 and cancer-related mRNA?
- How can the dynamic function of FXR1 in cancer development be overcome?
- What role does FXR1 play in the coordination of cancer phenotypes across various genetic backgrounds?
- How can we best target FXR1 as a molecular biomarker or therapeutic target in the field of tumor biology?

## Introduction

RNA binding proteins (RBPs) carry out a wide-ranging and critical function in RNA metabolism. RBPs interact with specific RNAs’ forming ribonucleoprotein (RNP) compounds. Hence, regulating gene expression processes like cleavage and polyadenylation, RNA splicing, export, stability, translation, and the degradation of coding- and non-coding- RNAs, as well as their precursors [1–5]. RBPs execute their role by interacting with proteins and various classes of RNA (mRNAs, snoRNA, snRNA, tRNAs, ribosomal RNAs, and non-coding RNAs), regulating their fate and functions [6, 7]. So far, Over 1500 RBPs have been discovered in the entire human genome using recent high-throughput screening, which accounts for ~7–8% of all proteins encoded by genes: though only a few of them have been functionally described [8]. RBPs have important functions in various stages of gene regulation; therefore, alterations in their expression or mutations can frequently affect various pathological and physiological processes that lead to diseases, including malignancies [9–12]. RBPs interact with their respective RNA targets via RNA binding domains (RBDs). Approximately 50 RBDs have been discovered, including RNA-interacting protein domain, zinc finger domain, PAZ domains, heterogeneous nuclear RNP K-homology domains, RNA recognition motifs, and dsRNA-binding domains, among others, which allow RBPs to be generically categorized [13].

Firm evidence exists that RBP dysregulation, specifically FXR’s occurs in various human cancers where it promotes tumorigenesis. *FXR* genes family encode very similar RBPs, like Fragile X mental retardation 1 (*FMR1*), *FMR1* autosomal homolog 1 (*FXR1*), and *FMR1* autosomal homolog 2 (*FXR2*), which have been identified on chromosomes Xq27.3, 3q26.33, and 17p13.1, respectively [13, 14]. Several cancer-related genes are up/downregulated by *FXR1* by modulating post-transcriptional and translational gene expression levels. *FXR1* dysregulation and mutations are associated with several disease pathogenesis, including cancer [10, 14]. FXR1 induces carcinomas in multiple tissues more than the other two proteins in the family. Many studies have found that FXR1 overexpression promotes carcinogenesis and inhibits senescence in squamous cell carcinoma (SCC) of the lung and head and neck squamous cell carcinoma (HNSCC), resulting in a poor prognosis and survival [15, 16]. Several studies have discovered that *FXR1* overexpression at both the RNA and protein levels is strongly associated with a poor prognosis in several cancers.

### Structure and function of FXR1

The sequencing of *FMR1* enabled the fast discovery of human *FXR1* (3q28) via sequence homology [14]. *FXR1* genes, unlike *FMR1* genes, are autosomal. FXR1 protein exhibits a similar structure to other family members, with 60% identical amino acid sequences; however, their C-termini vary from one another, suggesting that they have distinct roles [17]. The N-terminus of the FXRIP protein comprises Tandem agent-like domain followed by a non-classical nuclear localization signal (NLS), three protein K homology (KH) domain, a nuclear export signal (NES) and an RGG box at C-terminus. Due to their KH and RGG domains, FXR1 was classed as RBP. Apart from FXRI’s ability to bind to RNA, they interact with other members through the NTD domain, which is similar to the 40S and 60S ribosome subunits [18, 19]. NLS enhances FXR1-RNA interaction and affinity among FXRs [20]. Most FXR1 isoforms have nucleolar targeting signals (NoS) on their C-termini [21].

Exportin-1 shuttles FXR1 to the cytoplasm from the nucleus, although its shuttling depends on its isoform [21, 22]. RGG motifs of FXR1 bind to DNA or RNA structures such as G-quadruplex RNA and secondary RNA structures with four guanines; however, this binding affinity can be altered by targeted RNA arginine methylation [18, 23]. FXR1 KH domain binds targets RNA, and a single base change may affect FXR1 structures, which leads to disease development [24–26]. These interactions of FXR1 suggest that FXR1 co-regulates and affects RNA and target mRNA expression.

### FXR1 expression and localization

Although variations in expression levels among tissues, FXR1 is ubiquitously expressed. FXR1 is widely expressed in the heart, skeletal muscles, brain, and testes [26, 27]. Even in the same tissues, FXR1 is expressed differently across cellular compartments and cell types, demonstrating its targeting and function diversity [26]. FXR1 is present pre-synaptically in axonal regions of the brain’s Olfactory Blub, thalamus, and CA3 area [28, 29]. Within the cytoplasm, FXR1 is observed in ribonuclear particles, ribosomes, and RNA [18, 30, 31].

### Post-translational modifications

Since FXR1 plays numerous roles and exists in many cells, it is not surprising that they are regulated by different post-translational modifications (PTMs). Among these include sumoylation, ubiquitylation, acetylation, methylation, and phosphorylation [32, 33].

The association between phosphorylated FXR1 with RISC and polyribosomes represses the translation of target mRNAs. The well-studied FXR1 PTM is serine 420 phosphorylation (S420), which is probably regulated by the constitutive active casein kinase II (CK2) and essential for its involvement in translation regulation. Moreover, the recruitment of FXR1 to arsenite-induced stress granules increases this PTM, suggesting that it plays a role in cellular stress responses [34, 35]. Protein phosphatase 2A (PP2A) dephosphorylates FXR1 and releases it from ribosomes and AGO2, consequently promoting translation. It has been established that FXR1 phosphorylation at S420 is necessary for the development of distinct RNA granules involved in RNA transport and stress responses. The process of sumoylation probably triggers the release of FXR1 from RNA granules. Furthermore, FXR1 ubiquitylation and destruction by the ubiquitin-proteasome system (UPS) require dephosphorylation of S420 [32, 33]. By methylating the FXR1s RGG domains, their ability to form homo- and heterodimers with other FXPs and their association with polyribosomes may be compromised. While methylation of FXR1 has the potential to ultimately diminish the RNA-binding activity of FXR1 [32, 33]. Multiple more PTMs of FXR1 were discovered by proteome-wide techniques [36, 37], but their origins and effects have not yet been investigated.

### Mechanistic role of FXR1

#### FXR1 regulates miRNA processing

RBPs regulate miRNA biogenesis and maturation via regulating canonical proteins, including Drosha, Dicer, and RISC (miRNA-induced silencing complex). FXR1 protein has four RBDs: coiled-coil domain, three K Homology domains (KH1-2), NLS and a C-terminal domain including RNA-binding Arginine-Glycine-Glycine (RGG) domain. FXR1 proteins require these domains to recognize various miRNAs [38].

Post-transcriptional and transcriptional factors influence miRNA expression. FXR1 is essential for efficiently processing neuronal miRNAs and impacts pre-miRNAs’ processing, transport, and stability. In the DT40 cell line, Dicer knockdown enhances FXR1 expression and miRNA-mediated target silencing [39]. FXR1 increases brain-specific miRNA processing by raising mature miR-9 and possibly decreasing pre-miR-9-2. FXR1 forms complex with pre-miRNAs and Dicer and processes pre-miR-124 and pre-miR-9 in vitro. These findings suggest that FXR1 regulates brain-specific miRNA expression [40]. Translational suppression of specific mRNAs via the RISC is thought to be accelerated by the FXR1’s will-recognized interaction with the miRNA machinery [41, 42]. Gessert et al. found that FMR1/FXR1 regulates the stability and translation of Rx1, Pax6, and FoxD3 mRNA through binding to RISC and miRNAs (miR-130a, -219, -23b, -200b, -96, and -196a). FMR1/FXR1 depletion reduces mRNA expression impairs eye development and cranial cartilage formation [43]. FXR1 lack in skeletal muscle embryos reduces miR-1, a cardiovascular development-miRNA, by 45% [43], suggesting that FXR1’s role is not limited to neuronal miRNAs. Muscular defects may result from FXR1 deficiency-induced miR-1 dysregulation [44].

**Fig. 1:**
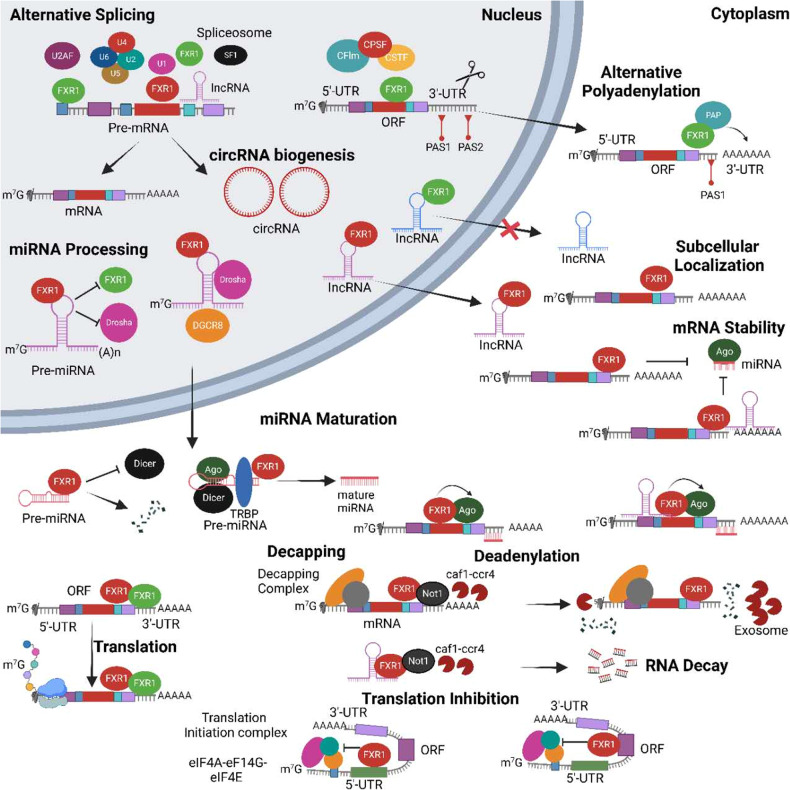
Biological functions of FXR1 in the post-transcriptional mechanism that regulates gene expression in cancer. They can define the future of an RNA by modulating multiple events, such as its miRNA-mediated processing, alternative splicing, alternative polyadenylation, subcellular localization, and stability. Illustrations depicting the functioning mechanisms of FXR1 are presented in schematic diagrams.

In mammals, miRNAs are degraded by miRNases such as *XRN1*, *XRN2*, *RRP41*, and *PNPT1* [45–47]. RBPs bind to miRNAs by forming miRNA complexes and protecting them from degradation. miR-144 is stabilized by RBPs ILF3 and BUD13, as does RBP QKI stabilize miR-20 by forming a complex [48], suggesting that RBP-miRNA interactions stabilize miRNAs. FXR1 inhibits *PNPT1’s* exonuclease activity by binding to miR301a-3p and protects it from exoribonuclease *PNPT1*-mediated degradation. The *PNPT1* 3′-5′ exonuclease activity is inhibited by the presence of RNA secondary structure or RBPs at the 3′-end [49]. Target-specific miRNA and AGO2 interact with FXR1 to regulate post-transcriptional gene expression. These findings reveal how mammalian FXR1 protein regulates the stability of mammalian miRNAs (Fig. 1).

#### FXR1 involved in the localization of RNAs

The mRNA and lncRNA stability and translation rely on subcellular localization, as the cancer-related RBPs frequently bind to the subcellular compartments where mRNA and lncRNA are localized and translated [50, 51]. FXR1 are new cytoplasmic RNA-binding proteins with basic architecture like FMRP and are associated with cytoplasmic RNPs [52]. The FXR1 protein family is involved in several RNA processes, such as the subcellular localization of RNA by cytoplasmic shuttling [13] and interaction with motor proteins [53–55]. FXR1 is linked to mRNP structures that contain poly(A)+ mRNA in polyribosomes that are actively translating. It has NLS and an export signal, making it a potential mRNA carrier (chaperone) to the cytoplasm from the nucleus [52].

Nuclear pore complexes (NPCs) within the nuclear envelope (NE) facilitate macromolecule transport across the nucleus and cytosol. At the end of mitosis and during interphase, nucleoporins (Nups) assemble into NPCs in mammalian cells. In the absence of FXR1 or microtubule-based transfer, Nups improperly localize to the cytosol and form cytoplasmic nucleoporin granules, impeding the potential of NPCs to export protein. Furthermore, considering the well-established functions in translation pathways, FXR1 may transport Nups to the NE and govern their translation during interphase [56–58].

RNA sequences and trans-acting elements regulate mRNA’s translation, stability, and localization in the cytoplasm [59]. The 3′-UTR of oncogenes, cytokines, and growth factors contains well-studied AU-rich elements (AREs), which may trigger immunological disorders and cancers [60, 61]. AREs regulate mRNA export and translation in addition to their role in decay (Fig. 1) [60, 62, 63].

#### FXR1 involved in the stability of the RNAs

The 3′-poly(A) tail and the 5′-terminal 7-methylguanosine (m7G) prevent mRNA from decay and promote translation initiation. Deadenylation of the poly (A) tail, 5′-3′ exonucleolytic decay, decapping of the 5′-terminal m7G cap, and exosome-mediated decay contribute to mRNA degradation. mRNA that is slated for degradation are transported to stress granules or P-bodies for degradation [64, 65].

RNA sequences and trans-acting elements regulate cytoplasmic mRNA localization, translation, and stability [59, 66]. The well-studied AREs in the 3′-UTR of cytokines, oncogenes, and growth factors may trigger immunological diseases and malignancies [60, 61]. In addition to being involved in mRNA decay, AREs regulate translation and export [60, 62, 63]. To stabilize mRNA, FXR1 associates with the miRNA complex or AREs through their KH domains [67, 68]. FXR1 and AGO2 bind to miR369-3 and Let-7, respectively, and inhibit translation during the cell cycle [69]. Even though FXR1 regulates the stability of *p21* mRNA via AREs within the 3′-UTR, it is not yet explicitly associated with the RISC pathway [70]. FXR1 binds to the AREs elements (AUUUA) in the 3′-UTR of *cMYC* mRNA and upregulates their protein levels [71]. FXR1 has also been shown to bind to numerous inflammatory mRNAs and reduce their stability [72]. Mechanistically, it was proposed that mRNA decay was caused by competition between the RNA-destabilizing FXR1 protein and the RNA-stabilizing HuR protein on ARE-containing transcripts. Previous research has shown that HAdV-infected cells benefit from HuR protein stabilization of canonical ARE-containing reporter gene transcripts [73]. As a result, we considered the possibility that ARE-containing transcripts could be the target of an FXR1-HuR interaction that regulates MLTU mRNA turnover.

Both myoblast proliferation and cancer quiescence are regulated by *p21* through FXR1 [74]. Some miRNAs suppress *p21* mRNA expression and accelerate cell cycle development and progression [74]. FXR1 is known to control the cell cycle in proliferating cells, such as neural stem cells (NSCs) [75]. FMR1/FXR1 depletion decreases *Rx1, Pax6, and FoxD3* mRNA expression through interactions with RISC and miRNAs (miR-130a, -219, -23b, -200b, -96, and -196a), resulting in aberrant eye development and cranial cartilage formation [43, 76]. FMRP/FXR1 regulates many synaptic proteins involved in NMDA receptor activation by interacting with *GluN1, GluN2A, and GluN2B* mRNA [77, 78]. Previous research has demonstrated that FXR1 regulates *p21* by interacting with the G-quadruplex (G4) RNA sequence in *p21* mRNA (Fig. 1) [79, 80]. We have recently identified that FXR1 overexpression destabilizes *PDZK1IP1* and *ATOH8* mRNA expression and promotes the development of esophageal cancer. Mechanistically, FXR1 negatively regulates *PDZK1IP1* or *ATOH8* transcripts by promoting mRNA degradation via direct interaction with its 3′-UTRs. Our findings show that FXR1 has oncogenic activities through the *PDZK1IP1*/*ATOH8* pathway, which might have potential diagnostic or therapeutic implications (unpublished data).

#### FXR1 involved in the regulation/translation of RNAs

mRNA translation is among the numerous posttranscriptional processes that are significantly impacted by RBPs [81]. Translation is the cellular activity that requires the most energy and precision in regulation, regulated by both cis-acting RNA elements like terminal oligopyrimidine motifs and CA-rich regions [82], and trans-acting factors like mRNA binding proteins (mRBPs) [81]. The latter impacts every stage of translation, whether it is transcript-specific or global [81]. A considerable number of mRBPs and (m)RNA binding domains have been identified via mRNA interactome capture’ techniques based on mass spectrometry [83, 84]. These approaches identify mRBPs physically linked to sequence- or structure-specific regions in mRNAs that influence gene expression at the posttranscriptional level.

FXR1 regulates mRNA translation efficiency in many ways. FXR1 exploits the miRNA pathway to inhibit translation initiation or elongation. Although the regulatory mechanisms are thought to be the same, the FXR family of proteins may have different targets. FMRP interacts with CYFIP1, binds to the translation-initiating factor eIF4E, and inhibits the translation-initiating complex assembly to regulate MAP1B, APP, and CaMKII levels in neurons [85, 86]. Since they bind to CYFIP2 rather than CYFIP1, it is not the exact process by which FXR1 operates; nonetheless, an alternative approach is possible [87]. Elongation stalling is the predominant translation regulatory mechanism through which FXR1 regulates translation. When phosphorylated, FXR1 interacts with polyribosomes since it binds to the entire open reading frame and the UTRs [88]. FXR1 regulates mRNA translation by interacting with miRNAs and the RNAi pathway through the RISC protein AGO2. AREs in the 3′-UTR of mRNA are key post-transcriptional regulatory signals that may modify mRNA translation and stability instantly, thereby modifying gene expression with clinical and physiological implications. The interaction of FXR1 with the 3′-UTR AREs regions of *cMYC* mRNA promotes translation. FXR1 enhances *cMYC* translation by binding to the 60S ribosomal subunit, promoting polysome accumulation, and maintaining the mRNA [71, 89].

*TNFα*-ARE regulates translation activation in human cell lines through FXR1 and AGO2. FXR1 form the FXR1/AGO2/microRNA complex interacts with DAP5/p97 in the quiescent state, promoting non-canonical translation. The distinct contributions of FXR1 and AGO2 in cell-growth-dependent translation activation give insight into ARE-mediated regulation and link two fundamental post-transcriptional regulatory mechanisms [90, 91].

Several studies have indicated that miRNAs act as ARE-containing mRNA regulators. First, RISC contains two ARE-associated proteins, PAI-RBP1 and FXR1 are ARE-associated [67]. Second, miRNAs and ARE-binding proteins share the same cytoplasmic bodies [92, 93]. Third, Tristetraprolin (TTP) through RISC via AGO2 and miR16-1 [94] regulates *TNFα* 3′-UTR levels.

FXR1, TTP family members, and HuR interact with *TNFα* and other AREs in response to cell signaling pathways to alter translation or stability [95–97]. Moreover, in response to *TNFα* translational upregulation, FXR1 inhibition induces muscle atrophy, reduced growth, and neonatal mortality [98]. According to another study, the FXR1 RGG motif binds with eIF4A1 and eIF4E to recruit the eIF4F complex to the cMYC translation initiation site. The lack of the RGG motif blocked FXR1’s interaction with the eIF4A1, eIF4E, and eIF4G1 proteins. Our finding shows that the RGG-box domain of FXR1 recruits eIF4F to the mRNA translation start site (Fig. 1) [71].

FXR1 has recently been identified as a new m^6^A reader protein [99]. Importantly, Price and coworkers [100] showed that knocking down METTL3 (an m6A catalytic enzyme) greatly decreased capsid protein accumulation and dramatically reduced infectious HAdV-5 progeny by reducing splicing of MLTU pre-mRNAs. Unexpectedly, they observed that the elimination of cytoplasmic m^6^A readers YTHDF1, YTHDF2, and YTHDF3 had a negligible impact on the infectious cycle of HAdV-5 in their investigation [100]. These findings imply that an additional cytoplasmic m^6^A reader protein, like FXR1, targets m^6^A-modified MLTU mRNAs and regulates their stability and translation. Endogenous FXR1 binds selectively to m^6^A-modified MLTU transcripts and protects the m^6^A signal on pVII and fiber mRNAs. These findings may suggest that FXR1 regulates the translation of mRNAs, especially in m^6^A-dependent manner.

#### FXR1 and defects in RNA editing

Interestingly, FXR1, in particular, is connected to RNA editing mechanisms. Exquisite studies in Drosophila found that FXR1 physically interacts with and regulates the activity of dADAR, an A-to-I- editing enzyme in RNA. Here, aberrant RNA editing was caused by deletion or overexpression of dFXR1, particularly of synaptic transmission and neuromuscular junction architecture-related mRNAs [101]. In humans, FXR1 interacts with the functional active A-to-I RNA editing enzyme ADAR1. The fact that differential RNA editing sites in several diseases are near FXR1-binding sites indicates that FXR1 plays an immediate role in the recruitment of ADAR enzymes to mRNA-editing sites. It is interesting to note that FXR1 may inhibit the editing of certain mRNA sites [102], probably impacting the spinal cord motoneurons in patients [103], which may have implications for synaptic transmission, the integrity of neuromuscular junctions, and motoneuron survival.

#### Other function of FXR1

In addition to its role in RNA metabolism, FXR1 also plays important roles in regulating chromatin dynamics and the DNA damage response, the cell cycle, ribosome biogenesis, and mitochondrial organization [32, 104, 105]. FXR1 has a key function in modulating ion channels at numerous stages in neurons. In addition to mediating the regional translation of mRNAs which encode for different ion channels, FXR1 also plays a vital function in the trafficking and gating of channels through interactions between proteins [106]. Furthermore, FXR1 plays a significant role in cellular stress responses and stress granule production. Stress granules are membrane-free assemblies of proteins and RNAs that form in the cytoplasm as a result of protein/RNA phase separation, and facilitate the majority of cell types to survive under stressful environments [107]. All three FXPs, like many other RBPs, are known to be found in stress granules [108], but FXR1 is particularly important for stress granule assembly [109]. Interestingly, the expression of FXR1 is induced by various stressors in different cell types, providing additional evidence for its involvement in cellular stress responses. It is worth noting that lower and greater levels of FXR1 expression are related to reduced and enhanced cell viability, respectively [110].

Recently, a study has demonstrated that FXR1 binds to proteasomes, and that proteasome activity increases in the absence of FXR1, indicating that FXR1 plays a significant role in proteasome regulation. Further evidence pointed to a role for it in intracellular ubiquitination. Because little was known about the association between FXR1 and the ubiquitin-proteasome system (UPS), these results are interesting and provide novel insights into FXR1’s functioning mechanism. Moreover, by reassessing FXR1 in the context of UPS, a novel understanding of the pathogenesis of diseases associated with FXR1 may be attained [111]. In FXR1-associated diseases, for instance, problems with protein quality control may be caused by FXR1 dysregulation. Future research and investigations are anticipated to reveal novel functional pathways of FXR1 as a UPS-related mediator in biological activities and disease.

### FXR1 modulation of cancer phenotypes

**Fig. 2:**
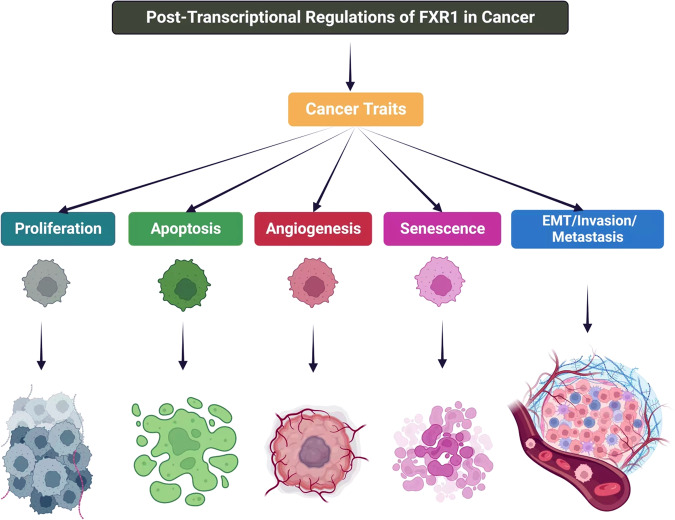
The role of FXR1 in cancer predisposition. FXR1 is an important part of the manifestation of various cancer hallmarks, including proliferation, apoptosis, angiogenesis, senescence, and EMT/invasion/metastasis.

**Fig. 3:**
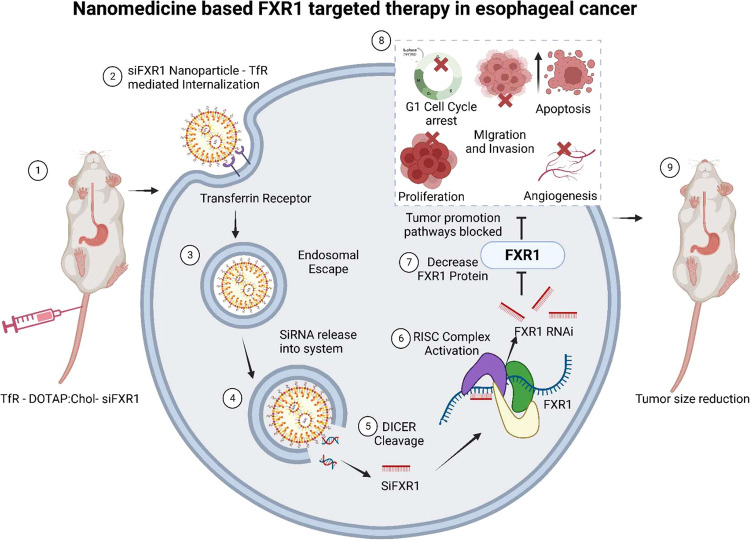
A schematic illustration depicts the use of nanomedicine to target FXR1 in esophageal cancer treatment. Image produced using BioRender.com.

FXR1 has been related to almost every step leading to tumor formation. This altered FXR1 activity seems to be present in all cancer types, and it appears to be associated with the dysregulation of the corresponding mRNA targets. An alternative way of thinking about FXR1 roles in cancer is to divide them into different categories: prolonged proliferative potential, evading cell death and senescence. Below are examples of FXR1 involved in each phase of these fundamental biological processes (Fig. 2) and (Table 1). Moreover, Fig. 3 schematically illustrates how nanoparticles can be employed to deliver drugs or siRNA that interfere with FXR1 expression or activity/stability in esophageal cancer cells.

**Table 1:** FXR1 overexpression in different cancers.

Cancer type	Expression in disease	Murine model used	Cases	Function Pathway/ Mechanism of action	Pathological status/ Function	References
HCC	Up	BALB/c nude mice	88	FXR1 promotes HCC proliferation, migration, and invasion by regulating SMAD2/3.	In HCC patients, increased FXR1 expression is associated with a worse prognosis.	[83]
UCB	Up	BALB/c nude mice	175	FXR1 regulates *TRAF1* mRNA stability by facilitating the recruitment of the CFIm25 and CFIm68 components to the *TRAF1* 3′-UTR for 3′ processing	FXR1 regulates *TRAF1* mRNA stability by facilitating CFIm25 and CFIm68 recruitment to the *TRAF1* 3′-UTR for 3′ processing.	[84]
Ovarian cancer	Up	Athymic nude mice	579	FXR1 binds to the AREs within *cMYC’*s 3′-UTR and stabilizes its expression.	*cMYC* is the primary target of FXR1’s cancer-causing effects on ovarian cancer.	[61]
prostate cancer	Up	-	-	FXR1 inhibited *FBXO4* transcripts through direct interaction with its 3′-UTR and promoted mRNA degradation.	FXR1 was found to be overexpressed at both the transcript and protein levels in prostate cancer cells.	[85]
Glioblastoma	Up	BALB/C athymic nude mice	3/3	FXR1 exhibited oncogenic effects through the *MIR17HG*/miR-346, miR-425-5p/*TAL1*/*DEC1* pathway.	FXR1 and *MIR17HG* were found to be overexpressed in glioma tissues and cells.	[86]
Cancers	Up	BALB/c nude mice	-	FXR1 not only binds RNA but also recruits the transcription factors *STAT1* and *STAT3* to the chromatin interface of gene promoters, where it controls transcription and so, at least in part, mediates cell proliferation.	Inhibiting FXR1 could be a promising therapeutic approach to treating human malignancies with *TP53* homozygous deletion.	[87]
oral cancer (HNSCC)	Up	nude mice	604	FXR1 overexpression promotes miR301a-3p-mediated *p21* suppression, which may occur in HNSCC to avoid senescence.	FXR1 can prevent *PNPT1*-mediated degradation of miR301a in HNSCC, resulting in *p21* suppression and contributing to the poor prognosis of oral cancer.	[88]
oral cancer (HNSCC)	Up	-	522	FXR1 overexpression binds to and destabilizes *p21* mRNA, lowering p21 protein production in oral cancer cells.	Overexpression of FXR1/*TERC* is associated with HNSCC proliferation and a poor prognosis.	[15]

#### Proliferation

Uncontrolled cell proliferation can cause a transformed cell population to expand and lead to cancer. A potentially effective cancer therapy must be able to stop or limit the unchecked proliferation of cancer cells. Most RBPs are involved in tumor cell proliferation, making them potential cancer progression drivers [112]. FXR1 is upregulated in numerous malignancies, suggesting it is involved in cancer development. FXR1 expression levels were increased in SCC of the lung, Non-small cell lung cancer, HNSCC, and prostate cancer [113, 114]. *FXR1* is an oncogene because it targets and stabilizes many cancer-related mRNAs, and its dysregulation in oncogenesis has a complicated molecular mechanism that varies depending on the cancer type. FXR1 regulates *p21* and *c-Myc* mRNA levels to promote cell proliferation [15]. FXR1 is also involved in regulating *TRAF1, FBXO4, COX2*, *TNF-α, eIF4E*-*BP2*, *CDK4*, *CCNE1*, *CCND1*, *CDK2*, and *CDK1* by binding to the 3′-UTR AREs [69, 115–124]. MiRNAs and ribonucleases promote cell proliferation by binding to their 3′-UTRs [123, 125, 126].

RBPs prevent miRNAs from degradation by forming miRNP complexes. RBPs QKI and ILF3 stabilize miR-20 and miR-144, suggesting that mRNA stability is associated with RBP-miRNA interactions [126, 127]. According to the TCGA analysis, patients with oral cancer who overexpressed FXR1 had poorer outcomes. FXR1 targets the tumor suppressor *p21* 3′-UTR, thereby promoting the progression of HNSCC and preventing cellular senescence. MiR301a-3p and FXR1 bind to the 3′-UTR of *p21* mRNA, promoting its degradation and accelerating the progression of HNSCC. In laryngeal squamous cell carcinoma (LSCC), miR301a-3p acts as a cancer-promoting gene, targeting multiple tumor suppressor genes, such as *Smad4* [128]. FXR1 specifically binds to miR301a-3p to form a miRNP complex that protects it from exoribonuclease *PNPT1*-mediated degradation, supporting the notion that it stabilizes miRNAs and enhances their oncogenic properties. FXR1 and miR301a-3p are upregulated in NSLSC and HNSCC cancers, suggesting a distinct regulatory mechanism for miR301a-3p stabilization by FXR1. Targeting FXR1-miRNA-mediated *p21* regulation can inhibit the growth and proliferation of oral cancer [15]. It is necessary to conduct additional research to investigate how the FXR1-miR301a-3p axis regulates the mRNA stability of other genes, which may drive cancer progression [15]. FXR1 knockdown alters the expression of several miRNAs in both positive and negative oral cancer cells, inhibiting the expression of miR301a-3p and miR29b-3p in different oral carcinoma cells. Through deadenylation, decapping, and degradation, both RBPs and miRNAs can target and modulate particular mRNA transcripts [129]. *CCR4-NOT* mRNA deadenylase complex, DCP1/2 decapping enzymes, and *XRN1/2* and *PNPT1* exonucleases have all been associated with mRNA decay mediated by sequence-binding proteins and miRNAs [130–134].

FXR1 is highly expressed in advanced and high-grade ovarian tumors. According to the clinical outcome study, FXR1 mRNA high expression is linked with decreased overall and recurrence-free survival [135]. CRISPR/Cas9-mediated deletions show that *FXR1*’s oncogenic effects require the AREs in the 3′-UTR of the *cMYC* gene. Overexpression of *cMYC* by FXR1 increased cell-cycle regulators such as cyclin E1, D1, and CDKs, promoting the growth of ovarian cancer. FXR1 promotes the translation of the *cMYC* oncoproteins by binding to the AREs within *cMYC* mRNA, which is essential for ovarian cancer progression and aggressiveness [71]. This demonstrates that *cMYC* mediates FXR1’s oncogenic effects, such as cell proliferation and metastasis.

FXR1 regulates NSC cell-cycle proteins (p21) during adult neurogenesis to maintain adult NSC proliferation [78]. FXR1 deficiency increases *p21* mRNA and its protein expression while inhibiting cell proliferation. This proliferation deficit can be reversed by restoring p21 to wild-type levels in NSCs. FXR1 promotes myoblast (muscle stem cell) proliferation by accelerating cell cycle progression and decreasing p21 mRNA stability [136, 137].

#### Epithelial-mesenchymal transition, invasion, and metastasis

During embryogenesis, tissue regeneration, wound healing, tumor progression, and metastasis, healthy cells undergo epithelial–mesenchymal transition (EMT). EMT and cell structure and function modifications are regulated by transcriptional and post-transcriptional gene expression. During EMT, FXR1 regulates mRNA translation, alternative splicing, polyadenylation, and stability [138]. In a loss of function study, FXR1 knockdown reduces hepatocellular carcinoma (HCC) cell invasion and migration via *TGF-β* modulation, whereas upregulation in HCC cells increases cell invasion which is abrogated by inhibiting SMAD2/3. *TGF-β-SMAD* signaling promotes metastasis and EMT of HCC. The EMT gene *Slug* is a popular *SMAD3* target [139]. Environmental stressors include reactive oxygen species, hypoxia, inflammation, and extracellular mediators such as epidermal growth factor, fibroblast growth factor-2, and *TGF-β* [140–144], which can cause EMT. FXR1 depletion inhibited *SMAD2/3* expression, whereas *SMAD2/3* knockdown decreased the production of EMT-related proteins [112].

#### Apoptosis

Cancer cells replicate and grow indefinitely, in addition to their ability to evade cell death. Apoptosis is a critical process that allows healthy cells to determine whether to die under extreme conditions. Cancer cells avoid apoptosis to accelerate tumor growth. RBPs regulate the expression of mRNAs involved in apoptosis, including *PARP*, *Bcl*, *p53*, *Fas*, *Caspases*, and others [145–148].

FXR1 is highly expressed in many cancers, including SCC of the lung and HNSCC [16]. Loss of FXR1 induces apoptosis and induces cellular Senescence in HNSCC [15].

George et al. 2021 investigated apoptosis in FXR1 knocked-out OVCAR5, Kuramochi, and HeyA8 cells. They observed that inhibiting FXR1 accelerates the death of these cells. Following TCGA datasets, GSEA results showed that FXR1 depletion upregulates mRNA levels of *CDKN1A* and *CDKN1B* while downregulates that of *CDK2*, *RAD51*, *BCL2L11*, *MCM2*, *CCNB1*, and *HMGA1*, all of which are essential genes in apoptosis functional annotation. *FXR1* deficiency may activate apoptosis biomarkers cleaved caspase-3 and caspase-7 [71].

FXR1 deletion decreased pro-survival proteins such as Survivin and HSP-60 while increasing pro-apoptotic proteins levels like cytochrome-C, BAX, and death receptors like p21, p27, FADD, phospho-p53, and FAS. FXR1 depletion decreases the levels of several oncogenic proteins, such as cyclin B1, cMYC, CHK1, EVI1, FOXM1, and CDC6, consistent with previous findings that FXR1 suppresses apoptosis and promotes oncogenesis. When FXR1 is depleted, the expression level of proteins BAX, p21, p27, FAK1, DUSP4, and PAI1 increases while BCL2 decreases, all of which are involved in cancer cell death pathways [71]. This finding shows that FXR1 silencing inhibits cancer cell growth, stops the cell cycle, and activates cell death pathways.

#### Senescence

Genetic changes, telomere length, reactive oxygen species, chemotherapy, and radiotherapy can all lead to cellular senescence in healthy and malignant cells. It is a permanent G1 cell cycle arrest characterized by metabolically active and viable cells. These cells typically activate the RB/p16 tumor suppressor pathways and the *p53*/*p21* stress response [149–151].

According to recent research, senescence suppresses tumors in vivo in premalignant tumors such as naevi, neurofibromas, and lung adenomas [152]. Most transcriptionally active genes, including *p21*, *p27*, *p16*, and *PTEN*, promote cellular senescence by activating *p53* or *p16*-mediated pathways [69]. Although transcriptional alterations affect cellular senescence, post-transcriptional modifications are poorly understood. During Senescence, RBPs and ncRNAs frequently interact to modulate gene expression post-transcriptionally [26]. In mammalian cells, many RBPs regulate mRNA processing, transport, translation, and stability of senescence-related genes. Because of their growing involvement in DDR, RBPs are the primary genomic instability regulators [153].

FXR1 has upregulated in HNSCC; inhibiting FXR1 causes deactivation of the phosphatidylinositol 3-kinase/Akt signaling pathway and induces the expression of genes associated with senescence such as *PTEN*, *p53*, *p21*, and *p27*. Overexpression of FXR1 regulates the cycle of *p21* and *TERC* mRNA to avoid senescence. FXR1 binds to and regulates *TERC* RNA, suppressing cellular senescence via telomerase activity. Deficient FXR1 in cancer cells activates *p53* causing DNA damage and eventually senesce. FXR1-mediated senescence is irreversible, and cells deficient in FXR1 cannot colonize or proliferate. FXR1’*s* unique *p53*-dependent regulation of *p21* expression inhibits cellular senescence in oral cancer cells [15].

FXR1 and miR301a-3p promote oral and lung cancer by decreasing *p21* expression. In FXR1-deficient cells, PNPT1 degrades miR301a-3p, increasing p21 protein translation and consequent oral cancer cell senescence [15]. miR301a-3p is an oncogene targeting many tumor suppressor genes, such as *Smad4* in LSCC [128].

### FXR1: a potential therapeutic target for cancer therapy

#### Drug resistance

Cancer treatment aims to eliminate tumors and improve patient survival. The complicated genetic landscape of most malignancies makes it challenging to get the optimum treatment responses and leads to therapeutic failure. Drug resistance is a complex issue and a significant factor in the failure of anticancer therapy [154]. It is mostly controlled by the tumor microenvironment, consisting of stromal population, immunological, and cancer cells. FXR1 and treatment resistance have been examined in pancreatic, breast, brain, and lung cancer [155]. FXR1 post-transcriptionally regulate multidrug resistance (MDR)-related genes, supporting them as therapeutic targets.

FXR1 decreased *KEAP* mRNA stability, and FXR1 knockdown caused cell death and limited axitinib resistance. Even in clear cell renal cell carcinoma (ccRCC) cells with downregulated FXR1, *KEAP1* knockdown increased apoptosis, suppressed autophagy, oxidative stress, and axitinib resistance. FXR1 altered the *KEAP*/*Nrf2* pathway, causing ccRCC cells to resist axitinib [156].

Through regulating CDC6, *FXR1* overexpression boosted tumor growth and oxaliplatin resistance in colorectal cells, while knockdown improved their sensitivity [157]. FXR1 knockdown reacts similarly to epirubicin. FXR1 knockdown increased ROS generation and ROS-FXR1-TGF-b-mediated epirubicin sensitivity. siFXR1’s galectin-3/b-catenin signaling suppression inhibited MDR protein expression in colorectal cancer cells [158]. Many studies have correlated FXR1 knockdown to enhanced radiation sensitivity. FXR1 upregulated caspase-2 and inhibited thioredoxin reductase in breast and colorectal cancer tissues, making them more radiation-sensitive. FXR1-mediated post-transcriptional modulation of ARID1A also increased radiation therapy resistance in breast cancer cells [159]. Finally, FXR1 in tumors affects treatment sensitivity and resistance. FXR1’s role in resistance and sensitivity may vary due to the genetic makeup of various malignancies. FXR1-based targeted therapy might have more options if the specific mechanism by which FXR1 influences treatment responses in animals and clinical studies is elucidated.

#### Approaches for targeting FXR1

**Fig. 4:**
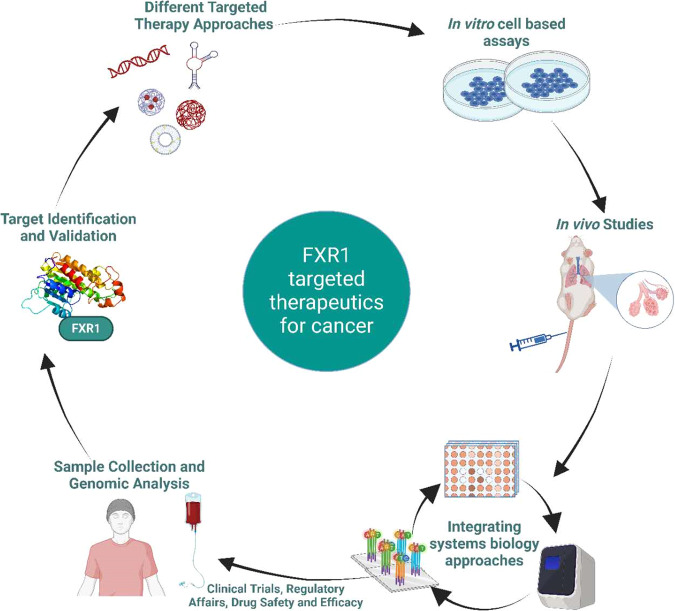
Presents an overview of the numerous pharmacological, biochemical, and nanomedicine-based FXR1 inhibition approaches that can be available for use in different cancer models. Image generated using BioRender.com.

**Fig. 5:**
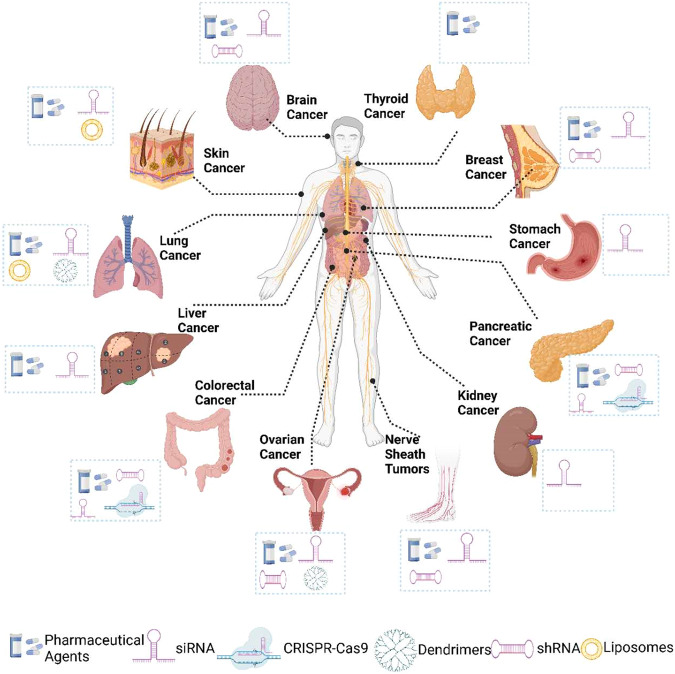
Schematic illustration of the translation of FXR1-targeted therapies from the bench to the bedside for cancers. Generated using BioRender.com.

As previously stated, FXR1 has been associated with tumor development and resistance to anticancer drugs. FXR1 is an attractive target for cancer treatment due to its widespread expression in nearly all cancers and its important function in the post-transcriptional controls of key genes involved in tumor growth and survival. Therefore, it is essential to develop new therapeutic approaches to block the biological actions of FXR1. Several inhibitors, like shRNAs or siRNAs, have been recommended to target *FXR1* mRNA, which is discussed briefly in the following sections. Inhibitors, antisense oligonucleotides (ASOs), Small-molecule medicines, and short therapeutic peptides are among the future treatment strategies that are being designed and examined in clinical trials [160–163]. Fig. 4 illustrates the diverse strategies that can interfere with FXR1 expression or activity in various cancer models. These strategies aim to modulate the interaction of FXR1 with its target RNAs and alter the expression of oncogenes and anti-oncogenes. However, Fig. 5 outlines the steps involved in the development and application of FXR1-targeted therapies for different cancers, from basic research to clinical trials. It also discusses some of the challenges such as reliable biomarkers, optimal drug delivery systems, drug resistance, and long-term outcomes, as well as some of the opportunities that need to be addressed in order to translate FXR1 research into effective and safe treatments for different cancer patients.

#### RNA interference-based approach

RNA interference (RNAi) lowers gene expression of disease-causing genes. They also modulate “druggable” targets and, more significantly, “non-druggable” targets that drugs or small-molecular inhibitors cannot regulate. RNAi affects cells in two ways. The first adds commercially produced siRNA to the biological system and then integrates it into the RISC complex. Once the siRNA guide strand binds to its complementary strand, the Argonaute proteins cleave the target mRNA to shut down the gene expression [164].

shRNA and siRNA were used to inhibit FXR1 in mice ovarian cancer cells. In ovarian cancer cells, transient and constitutive FXR1 inhibition by shRNA and siRNA reduced cell proliferation, colony formation, migratory, and invasion ability and suppressed tumor development in vivo. Instead of inducing tumor cell death, this decreased cancer cell proliferation. In addition, si*FXR1* therapy reduces *cMYC* and *Ki67*, the primary target of FXR1s. In tumor tissues, si*FXR1* administration upregulates *p21*, *p27*, and cleaved caspase-3. Hence, FXR1 may be a promising target for *cMYC* reduction in ovarian and other cancers. FXR1 siRNAs in nanoliposomes composed of 1,2-Dioleoyl-sn-glycero-3-phosphocholine (DOPC) reduced ovarian cancer growth and metastasis. These findings suggest that targeting FXR1 to lower its expression level through siRNA/shRNA reduces cancer cell growth, proliferation, and metastasis.

#### Genome editing approaches

CRISPR-Cas9 system for genome editing is a widespread method for genetic alteration. This approach examines gene function using precise genomic editions. The utility of CRISPR-Cas9 technology in diabetes, obesity, neurodegenerative, and ophthalmic illnesses is supported by data [165–167]. CRISPR-Cas9 suppresses the *FXR1* gene expression (U2OSFFF and delACAG) in several cancers. In vitro, FXR1 knockdown led to a decrease in malignancy, higher apoptosis rate, and lower sphere formation than wild-type cells, indicating their significance in cell proliferation and growth. FXR1 deletion in mice reduced tumor development by down-regulating oncogenic gene expression. Others have verified that *FXR1* depletion using CRISPR-Cas9 is beneficial in cancer treatment [160].

#### Nanomedicine-based approaches

Several studies have shown that employing nanomedicine-based techniques has a considerable advantage over traditional therapies. Some of the most frequent nanomaterials employed in the treatment of cancer include liposomes, metallic nanoparticles, polymeric nanoparticles, dendrimers, and solid–lipid nanoparticles [168, 169]. Different targeting ligands have previously been utilized to adorn the surface of nanoparticles to investigate the effective targeting of nanocarriers. To achieve targeted siRNA distribution and effectively execute mRNA suppression, conjugating siRNA to nanocarriers like dendrimers and liposomes would help minimize siRNA degradation [170]. Recently, researchers have focused on the use of extracellular vesicles (EVs), dendrimers, liposomes, and ASOs to improve FXR1s siRNA/shRNA distribution in cancer models [171, 172].

#### Extracellular vesicles (EVs)

Various techniques are now available for producing and characterizing EVs. EVs may also be utilized as biomarker molecules or potential candidates for liquid biopsies of clinical disorders since their composition is substantially impacted by the cell type from which they arise [173, 174]. When isolated from various cell types, tissues, and body fluids (including amniotic fluid, serum/blood, breast milk, saliva, and urine), there is evidence that EVs may be utilized to detect diseases [175, 176]. Natural drug carriers influence cell-cell communication by crossing physiological barriers, including the blood-brain barrier [177], while conventional drugs cannot access them. Because of these properties, EVs are important carriers to target FXR1 for cancer treatment and diagnostics.

#### Antisense oligonucleotides (ASOs)

Antisense oligonucleotides have been used in cancer therapies in the context of drug delivery and angiogenesis [178]. *FXR1* has been implicated in inflammation by altering the expression of important pro-inflammatory molecules such as TNF-a, IL-1b, and IL-6. Therefore, suppression of FXR1, specifically using phosphorothioate phosphate-modified ASOs can be applied to reduce disease severity, at least in mice models. To improve cellular uptake and stability, ASOs may be incubated with cationic lipids (DOTAP) and administered through intranasal and intrathecal channels.

FXR1 expression is high in various human malignancies, including cervical, SCC of the lung, and HNSCC [71]. As a result, FXR1-inhibiting therapeutic interventions may have widespread applicability to other malignancies that contain *FXR1* CNV. An unbiased genome-wide study examined the possibility that FXR1 can directly regulate numerous additional mRNAs as its targets. The use of pro-senescence techniques in the treatment of cancer is a promising alternative to conventional chemotherapy treatments [179]. FXR1 has the potential to function as diagnostic and prognostic markers for several malignancies due to their aberrant expression and mRNA regulatory functions. For instance, FXR1 is significantly overexpressed in the majority of cancers and is linked to an aggressive phenotype or a bad prognosis [180].

Furthermore, a high FXR1 expression level is associated with more severe features and worse survival outcomes in ductal breast cancer patients [159]. In the absence of FXR1, *PNPT1* degrades matured oncogenic miR301a-3p. In addition, it has been shown that *p21* is a target of miR301a-3p and that when FXR1 is knocked out, miR301a-3p is downregulated while *p21* mRNA and protein expression levels are elevated in different oral cancer patients. The downregulation of p21 signaling in an HNSCC cohort with overexpressed FXR1 and miR301a-3p might be explained by this pathway. According to these findings, FXR1 inhibitors combined with anti-miR oligonucleotide intervention and chemotherapy could be an effective therapy for HNSCC patients [15]. Besides, research is being conducted to classify RBPs as diagnostic and predictive biomarkers that reveal cancer-specific expression using modern bioinformatics methods such as microarray or newly processed RNA-seq data from the TCGA data [181, 182].

## Conclusion and future prospective

The regulation of RNA metabolism is an essential component of gene expression because it allows for the fine-tuning of transcript levels under physiological circumstances as well as the quick and dramatic changes in global gene expression associated with inflammation and immune responses. Long-term dysregulation of RNA metabolism is often associated with disease states, including cancer. FXR1 protein has emerged as an essential regulator of several aspects of RNA metabolism, with significant therapeutic potential.

FXR1 participates in almost every stage of post-transcriptional regulation, determining the fate and function of each transcript within the cells and maintaining cell equilibrium. They create ribonucleoprotein complexes that control RNA splicing, translation, localization, stability, polyadenylation, and degradation through dynamic interactions with different proteins and coding RNAs and ncRNAs [183]. This now becomes obvious that FXR1 is dysregulated in various types of cancers, affecting the production and functioning of cancer-causing and tumor-suppressor proteins. Therefore, deciphering the intricate relationships between FXR1 and its RNA targets associated with cancer could help improve our understanding of tumor development and possibly lead to the discovery of novel targets for cancer therapy [10]. According to the information in hand, FXR1 primarily affects the occurrence of cancer following a major carcinogenic activity by influencing many cancer-related downstream targets, thereby enhancing the biological implication of the initial transforming hit(s) via a “ripple effect”. In this case, *FXR1* predominantly functions as amplifiers in oncogenic driver mutation [184].

A lot of outstanding research leaves little question regarding the role of FXR1 in cancer etiology. Here we have the strongest evidence linking FXR1 to carcinogenesis, from studies of human tissue, animal models, and mechanistic investigations. Furthermore, there are several suggestions in the literature that the FXR1 may have a role in cancer associated with *cMYC*, *PNPT1*, *p21*, *p27*, *TERC*, and *p53*. The number of potential FXR1-binding oncogenes/tumor suppressor mRNAs might increase this list even more. Since most, if not all, patients share similar disease processes, dysregulated expression and/or function of the FXR1 may constitute a pathogenic event in cancer. Mutations in various genes are expected to have distinct effects on FXR1, although they may all lead to the same result. Therefore, it is more probable that loss of function and/or expression of FXR1 in cancer reflect one of the several “hits” required for cancer development. FXR1 deserves to be in the limelight and shift from stand-in roles to regular participants in carcinogenesis, given its scaffolding ability in developing RNP networks that regulate the expression of transcripts encoding proteins implicated in malignant procedures [10]. Numerous studies have repeatedly emphasized that FXR1 could be a useful biomarker for predicting the prognosis and treatment response of cancer patients. Few studies have demonstrated that small-molecule inhibitors or oligoribonucleotides can be used in vitro to selectively inhibit FXR1 or FXR1–RNA interactions, as previously established for HuR and LIN28, with positive functional outcomes [185, 186].

Due to recent advancements in employing more biologically accurate cellular models, such as patient-derived tumor xenografts, biomimetic microfluidic culture methods, and human tissue organoids, the cancer RBPome will soon be studied in unprecedented detail. In addition, these models will help in the identification of altered signaling combined through FXR1 in cancer and assist in identifying altered signaling pathways and PTMs that regulate FXR1. In the meantime, the development of synthetic FXR1s as molecular weapons is getting closer to being a reality. To control the regulation of individual or functionally related sets of cancer-associated transcripts with common recognizing patterns, these drugs might be designed to integrate distinct effector domains with particular RBDs [187]. Therefore, a more thorough investigation is needed to ascertain the specific role of the FXR1 and establish customized approaches for targeted FXR1 cancer therapy strategies without damaging nearby healthy cells. In addition, drug delivery systems should be optimized for target specificity to maximize the advantages of the identified drugs and enable future therapeutic applications of these different approaches [188, 189].

FXR1 is the key molecule that regulates the progression of cancer. Its transient or stable inhibition can significantly decrease cell survival and tumor development in vitro and in vivo through inhibitors such as siRNAs, shRNAs, ASOs, small molecules, and CRISPR/Cas9. These FXR1-targeting therapies based on RNAi, gene edition, and pharmacologic inhibition show considerable therapeutic effects in pre-clinical models; however, there are several challenges that must be overcome before they can be successfully implemented in clinical settings for the benefit of patients. First, the route of administration has a significant impact on the effectiveness of siRNA delivery. Second, siRNAs’ small size, short half-life, negative charge, difficulty in penetrating cell membranes, instability in the bloodstream, and susceptibility to nuclease degradation are the primary factors limiting their ability to travel to a particular target site. Thirdly, establishing cell- or tissue-specific delivery is an additional key impediment to the therapeutic application of siRNAs. Finally, genome editing using CRISPR/Cas-9-based technology in a therapeutic setting also confronts significant challenges. Its use is presently restricted due to restrictions on site-specific CRISPR/Cas-9 system delivery with minimal off-target implications. Therefore a recent study proposes using novel nanomedicine-based drug delivery approaches to address the issues associated with siRNAs and CRISPR/Cas-9, including off-target effects, site-specific delivery, and degradation. Designing efficient carrier molecules that facilitate site-specific delivery with little damage is critical to translate shRNA, siRNA, and CRISPR-Cas9-based strategies successfully.

In conclusion, we must gain a deeper understanding of FXR1 in cancers because they indicate great promise as potential therapeutic targets in the foreseeable future for cancer treatment. In contrast to targeting the expression of altered genes using antisense oligonucleotides or other approaches, targeting FXR1 could lead to shifts in the choice of therapy for the majority of patients, even those who do not have a genetic mutation in a commonly associated gene. Although the possibility of curing the respective disease through the restoration of FXR1 function and/or expression is unlikely, any advancement in currently available therapies is considered a success. We hope that in-depth new research will improve the applicability of developing FXR1-based diagnostic and treatment techniques in the coming days. This review might help to accelerate the expansion of FXR1 research and therapeutic applications in cancer clinics.

## Data Availability

All data generated or analyzed during this study are included in this published article.
